# Serologic, Virologic and Pathologic Features of Cats with Naturally Occurring Feline Infectious Peritonitis Enrolled in Antiviral Clinical Trials

**DOI:** 10.3390/v16030462

**Published:** 2024-03-17

**Authors:** Brian G. Murphy, Diego Castillo, N E Neely, Amir Kol, Terza Brostoff, Chris K. Grant, Krystle L. Reagan

**Affiliations:** 1 Department of Pathology, Microbiology and Immunology, School of Veterinary Medicine, University of California, Davis, CA 95616, USA; 2 School of Veterinary Medicine, University of California, Davis, CA 95616, USA; nneely@ucdavis.edu; 3 Custom Monoclonals International, 813 Harbor Boulevard, West Sacramento, CA 95691, USA; 4 Department of Medicine and Epidemiology, School of Veterinary Medicine, University of California, Davis, CA 95616, USA

**Keywords:** cat, FIP, feline coronavirus, antiviral compound, serology

## Abstract

Feline infectious peritonitis (FIP) is a multisystemic, generally lethal immuno-inflammatory disease of domestic cats caused by an infection with a genetic variant of feline coronavirus, referred to as the FIP virus (FIPV). We leveraged data from four different antiviral clinical trials performed at the University of California, Davis. Collectively, a total of 60 client-owned domestic cats, each with a confirmed diagnosis of naturally occurring FIP, were treated with a variety of antiviral compounds. The tested therapies included the antiviral compounds GS-441524, remdesivir, molnupiravir and allogeneic feline mesenchymal stem/stroma cell transfusions. Four client-owned cats with FIP did not meet the inclusion criteria for the trials and were not treated with antiviral therapies; these cats were included in the data set as untreated FIP control cats. ELISA and Western blot assays were performed using feline serum/plasma or ascites effusions obtained from a subset of the FIP cats. Normalized tissue/effusion viral loads were determined in 34 cats by a quantitative RT-PCR of nucleic acids isolated from either effusions or abdominal lymph node tissue. Twenty-one cats were PCR “serotyped” (genotyped) and had the S1/S2 region of the coronaviral *spike* gene amplified, cloned and sequenced from effusions or abdominal lymph node tissue. In total, 3 untreated control cats and 14 (23.3%) of the 60 antiviral-treated cats died or were euthanized during (13) or after the completion of (1) antiviral treatment. Of these 17 cats, 13 had complete necropsies performed (10 cats treated with antivirals and 3 untreated control cats). We found that anticoronaviral serologic responses were persistent and robust throughout the treatment period, primarily the IgG isotype, and focused on the viral structural Nucleocapsid and Membrane proteins. Coronavirus serologic patterns were similar for the effusions and serum/plasma of cats with FIP and in cats entering remission or that died. Viral RNA was readily detectable in the majority of the cats in either abdominal lymph node tissue or ascites effusions, and all of the viral isolates were determined to be serotype I FIPV. Viral nucleic acids in cats treated with antiviral compounds became undetectable in ascites or abdominal lymph node tissue by 11 days post-treatment using a sensitive quantitative RT-PCR assay. The most common pathologic lesions identified in the necropsied cats were hepatitis, abdominal effusion (ascites), serositis, pancreatitis, lymphadenitis, icterus and perivasculitis. In cats treated with antiviral compounds, gross and histological lesions characteristic of FIP persisted for several weeks, while the viral antigen became progressively less detectable.

## 1. Introduction

Feline infectious peritonitis (FIP) is a generally fatal coronaviral disease of cats caused by genetic variants of feline coronavirus (FCoV). FCoV manifests as two closely related biotypes—the relatively apathogenic feline enteric coronavirus (FECV) and the virulent FIP virus (FIPV) [1,2]. The relatively common FECV biotype replicates in feline intestinal enterocytes and is transmitted from cat to cat via the fecal–oral route [3,4]. According to the internal mutation hypothesis, the disease FIP is the result of genetic mutations in FECV, leading to a switch in viral host cell tropism from enterocytes to peritoneal-type macrophages, facilitating systemic spread and immunoinflammatory syndrome [5,6]. FIP is a complex, multi-systemic immuno-inflammatory disease characterized by fever, pyogranulomatous perivasculitis, effusion accumulation, fibrinous exudate and high mortality [3,4,5]. As a result of extensive systemic dissemination, FIP presents with clinical signs reflecting inflammation in multiple anatomic sites, including the abdominal cavity and associated viscera, thoracic cavity, central nervous system and/or eyes [5,6,7,8].

FIP presents in two classic forms, the effusive “wet” form featuring cavitary effusions or the granulomatous “dry” form, generally lacking effusions [2,7]. The two coronaviral biotypes are further categorized into serotype I and II viruses, with serotype II arising from a historic recombination event between FCoV and canine coronavirus (CCV) [8,9]. This genetic recombination resulted in the stable incorporation of the CCV *spike* gene within the FCoV genome [10]. Some investigators have argued that these two coronaviral serotypes are better interpreted as two distinct viruses [11]. The vast majority of naturally occurring FIP cases are associated with serotype I infections [12].

Spectacular advances have been made in antiviral therapies for ebolavirus, retroviruses, hepatitis C and human coronavirus infections [13,14,15,16,17]. Many of these therapies specifically target the function of viral nonstructural gene products like viral polymerase or protease proteins. The closely related nucleoside analogs GS-441524 and remdesivir (Gilead Sciences, Foster City, CA, USA) have demonstrated clinical efficacy in treating cats with FIP [18,19,20]. The nucleoside analog molnupiravir (EIDD-2801, Merck, New York, NY, USA) has been shown to effectively inhibit FIPV replication in in vitro assays [21,22] and has demonstrated clinical efficacy in cats with naturally occurring FIP [23]. Mesenchymal stem/stromal cells (MSCs) are promising candidate therapeutics for viral infections given their potent anti-inflammatory and immunomodulatory properties. Specifically, MSC treatment in chronic viral infection disease models induced a balanced and effective anti-viral immune response in concert with decreased systemic inflammation [24,25].

Since the discovery of effective antiviral compounds, thousands of cats with clinical signs of FIP have purportedly been treated worldwide [26]. Most of these cats, however, were directly treated by their caretakers and were not involved in hypothesis-based peer-reviewed clinical studies. A peer-reviewed retrospective study described the outcome of 393 caretaker-treated FIP cats using a survey-based method; serologic, pathologic and virologic data were not reported [27]. Although the outcomes of several prospective clinical trials involving antiviral therapies for FIP have been published, these studies have generally focused on the clinical data (clinical pathology data, cat behavior, quality-of-life score, body weight) and the ultimate ratio of clinical success to failure [19,23,26]. A few peer-reviewed reports have provided some pathology and virology data for antiviral-treated cats with FIP [18,20,28,29]. One study described a decrease in viral loads in blood, cavitary effusions and feces in response to treatment with GS-441524 [30]. Another study provided long-term (1 year) serology and virology data for feces and blood for GS-441524-treated cats with FIP [31]. A single study described the postmortem findings of a cat with FIP that was successfully treated with GS-441524 and in clinical remission 164 days before an unrelated death resulting from a road traffic accident [32].

Here, we report the serologic, virologic and pathologic features of cats with naturally occurring FIP associated with multiple antiviral clinical trials involving the administration of GS-441524, remdesivir, molnupiravir or MSC-based therapies. The detailed results of one of these trials, determining the efficacy of oral remdesivir compared to GS-441524, have been published [20]. Publication of the results for the other three clinical trials are pending. The virology, serology and pathology data presented here include cats that achieved antiviral-associated disease remission and those that succumbed to disease.

## 2. Materials and Methods

### 2.1. Clinical Trial Protocol

Four prospective, longitudinal clinical trials were performed on cats diagnosed with naturally occurring FIP. To be considered for enrollment, a diagnosis of FIP had to be obtained through (1) detection of the FCoV antigen by immunohistochemistry or immunocytochemistry in the context of granulomatous inflammation as interpreted by a veterinary pathologist or (2) the detection of FCoV RNA within effusion or tissue specimens. Additionally, the cat must have demonstrated ≥3 of the following clinical or clinicopathologic features: fever documented on two occasions over 12 h apart (rectal temperature > 102.5 °F), lymphocytes below the lower limit of normal, globulins above the upper limit of normal, albumin/globulin ratio < 0.6, bilirubin above the upper limit of normal, magnetic resonance imaging consistent with CNS FIP lesions as determined by a veterinary radiologist, ocular lesions consistent with FIP as determined by a veterinary ophthalmologist, effusion with total protein > 3.5 g/dL with cytology assessed by a veterinary clinical pathologist to be consistent with FIP, and positive FCoV antibody serology tested through a commercial laboratory or the UC Davis Clinical Diagnostic Laboratory. Cats were enrolled with caretaker-provided informed consent and with approval of the University of California Institutional Animal Care and Use Committee: protocol numbers 22327, 22773 and 23282. All cats were treated with antiviral therapy according to the individual study protocol. 

The different antiviral trials included effusive disease treated with GS-441524 with or without allogeneic adipose tissue-derived MSCs (Trial I), effusive disease treated with GS-441524 or remdesivir (Trial II), dry disease (non-effusive abdominal, thoracic, neurological and/or ocular FIP) treated with either GS-441524 or remdesivir (Trial III) and effusive disease treated with molnupiravir (Trial IV). The treatment arms included GS-441524 (effusive disease 12.5–15 mg/kg PO q24; dry disease 18–22 mg/kg PO q24), remdesivir (effusive disease 25–30 mg/kg PO q24; dry disease 38–42 mg/kg PO q24), molnupiravir (effusive disease 10–15 mg/kg PO q12) and a combination of GS-441524 (effusive disease 12.5–15 mg/kg PO q24) and two MSC infusions (below), with dose adjustments based on weekly weights reported by caretakers. All cats were treated for 12 weeks and monitored through periodic physical examination, owner assessment and blood chemistry/CBC for at least 16 weeks. A table defining antiviral trial assignment, the samples derived from each cat and the overall outcome is available in the Supplementary Materials.

Allogeneic adipose tissue-derived MSCs were isolated from a single specific-pathogen-free cat by the Regenerative Medicine Laboratory at the William T Pritchard Veterinary Medical Teaching Hospital (VMTH), UC Davis, as previously described and following an approved IACUC protocol 22327 [33,34]. Cryopreserved cells at low passage (P2–3) were thawed and cultured for 3–4 days and passed stringent quality assurance criteria before each treatment. In addition to antiviral therapy, five cats received two intravenous infusions, 2 weeks apart, with 20 million MSCs delivered as a slow direct injection over 20–40 min. All cats were hospitalized overnight post MSC-treatment to monitor for adverse reactions.

### 2.2. Handling and Storage of Feline Samples

Feline plasma, serum and effusion specimens were acquired from cats with active FIP. A positive control plasma specimen was obtained from a historic experimentally FIPV-infected cat [19]. Plasma was also acquired from specific-pathogen-free (SPF) cats for use as a negative control (UC Davis Feline Nutrition Colony). Feline plasma or serum samples were isolated by centrifugation from anticoagulant-treated whole blood (EDTA or heparin) and archived at −80 °C until they were utilized for Western blot or enzyme-linked immunosorbent assays (ELISA), as described below. Feline effusion specimens were also archived at −80 °C until they were utilized for these assays. Effusion cell pellets and tissue collected at necropsy were stored in RNAlater Stabilization Solution (Invitrogen, Waltham, MA, USA) and stored at −20 °C.

### 2.3. Immunoblotting of Feline Plasma and Ascites Fluid against FIPV Proteins

Feline serum or plasma and ascites fluid were assessed for immunoglobulin specificity to FIPV serotype I and II antigens and FIPV serotype II antigen-specific IgG, IgA and IgM.

Serotype I (Black I) and II (WSU 79-1146; GenBank DQ010921) FIPV were propagated in tissue culture as described previously [35]. Briefly, 3 × 10^6^ CRFK cells infected with FIPV serotype II or 3 × 10^6^ FCWF-4 CU cells infected with FIPV serotype I were rinsed 3 times with PBS, and then, lysed using an ultrasonic homogenizer (Sonic Dismembrator Model 100, Fisher Scientific, Hampton, NH, USA) with 100 µL of lysis buffer and 1 µL of protease inhibitor complex. The lysate was incubated for 30 min on ice, and centrifuged at 20,817× *g* for 10 min at 4 °C. The supernatant was transferred to new 500 µL tubes. The protein concentration of the supernatant was quantified with a BCA Protein Assay Kit (Prometheus), per the manufacturer’s instructions, using a microplate assay. 

A Western blot (immunoblot) was performed with a 4–12% precast SDS-PAGE gel (ab139596, Abcam, Cambridge, MA, USA) and 1 × Run Buffer (ab119197, Abcam, Cambridge, MA, USA). Sample wells were loaded with 12.5 µg serotype I and 12.5 µg serotype II viral protein suspended in 5 µL LDS sample buffer (ab119196, Abcam, Cambridge, MA, USA) and 2 µL DTT reducer in a total volume of 20 µL (ab119199, Abcam, Cambridge, MA, USA). 

The gel was transferred to a PVDF membrane in the transfer apparatus overnight at 4 °C. Membranes were cut into strips, washed in TBS-T (TBS buffer containing 0.1% Tween 20) three times and incubated in blocking buffer (20 mL TBS-T with 1 g 5% nonfat dry milk) for 1 h at room temperature. Strips were washed, and 3 mL of antibody dilution buffer (TBS-T with 5% bovine serum albumin) per strip was added to the containers. Effusion and plasma from study cats were added at 1:1000 to individual strips. Positive control FIPV3-70 and CCV monoclonal antibodies were added to two strips at 1:500 (Custom Monoclonals International, Sacramento, CA, USA). A negative control serum specimen at 1:1000 from an SPF was added to 1 strip. The strips were then incubated overnight at 4 °C on rocker apparatus.

The strips were washed and incubated in fresh antibody dilution buffer, and a secondary antibody was added at a 1:5000 dilution. Goat anti-mouse IgG-HRP (cat #1705047, BioRad, Hercules, CA, USA) was used as the secondary antibody for FIPV3-70 FIP and CCV, and goat anti-cat IgG(Fc) JD-HRP (cat# AAI26P, BioRad, Hercules, CA, USA) for feline plasma or effusion. The strips were incubated on a rocker for 1 h at room temperature, washed as described previously, exposed to 200 µL per strip of luminol and peroxidase (Prometheus ProSignal Femto kit) at a 1:1 ratio and incubated for 1–2 min based on the strength of signal desired. The strips were arranged in a plastic sleeve to recreate the shape and order of the original membrane and imaged with a digital imager (Analytik Jena UVP ChemStudio).

### 2.4. Enzyme-Linked Immunosorbent Assays (ELISA)

FIPV-infected cells were lysed and the protein quantified with a BCA Protein Assay Kit (Prometheus), per the manufacturer’s instructions, using a microplate assay. A 96-well ELISA plate was coated with 250 ng of viral protein per well. Effusion and plasma from study cats were diluted at 1:800. FIPV3-70 FIP/CCV monoclonal antibodies (1:500 dilution) were used as positive controls, and plasma from an SPF cat (1:800 dilution) was used as a negative control. One hundred microliters of primary antibody dilution was added to each well and incubated for 2 h at room temperature. Next, 100 µL of secondary antibody (1:3000 dilution) was applied, using goat anti-cat IgG(Fc) JD-HRP for the study cat specimens and the negative control and goat anti-mouse IgG-HRP for the FIPV3-70 FIP/CCV positive control. The plate was incubated at room temperature for 1 h. One hundred microliters of room-temperature single-component TMB Peroxidase ELISA substrate (cat# 1721068, BioRad) was added to each well. The plate was covered in foil and incubated at room temperature for 10 min, until sufficient blue chromogen developed within the positive control wells. The reaction was stopped with 50 microliters of 0.5 M H_2_SO_4_ per well. The absorbance of the plate was read with a spectrophotometer plate reader (SpectraMax 340, Molecular Devices) at wavelengths 450 nm and 595 nm.

### 2.5. ELISA and Western Blots for Anti-FIPV Immunoglobulin Isotypes

FIPV serotype II sample preparation, electrophoresis, transfer, blocking and primary antibody exposure were conducted as described above for each study specimen. To differentiate Ig subtypes, strips were exposed to one of the following secondary antibodies at a 10 µg/mL dilution: CM7 anti-IgM monoclonal antibody (Custom Monoclonals International, Sacramento, CA, USA), IgA9-6A anti-IgA (Custom Monoclonals International) monoclonal antibody or FDGI-2A1 anti-IgG monoclonal antibody (Custom Monoclonals International). A fourth group of control strips were not exposed to secondary antibodies. Strips were incubated in TBS-T on a rocker at room temperature for 1 h, washed as described previously and exposed to a tertiary antibody at a dilution of 1:3000. Strips exposed to a secondary antibody had a goat anti-mouse IgG-HRP tertiary antibody applied, and the unexposed control strips had a goat anti-cat IgG(Fc) JD-HRP tertiary antibody applied. The strips were incubated, washed exposed to luminol and peroxidase, and imaged as described previously.

Preparation of the ELISA plate using serotype II FIPV, blocking and primary antibody exposure were conducted as described previously. Nine wells were used per primary antibody. Secondary antibody dilutions were generated at 10 µg/mL using CM7 anti-IgM monoclonal antibody, IgA9-6A anti-IgA monoclonal antibody and FDGI-2A1 anti-IgG monoclonal antibody. Next, 100 microliters of the appropriate secondary antibody was added to each well, such that each primary-and-secondary antibody combination spanned three wells. The plate was incubated at room temperature for 1 h and washed in PBS-T. The wells that received a secondary antibody were exposed to 100 µL of goat anti-mouse IgG-HRP at a dilution of 1:3000. Unexposed wells received goat anti-cat IgG(Fc) JD-HRP at a dilution of 1:3000. The plate was incubated, washed, developed and imaged as described previously.

### 2.6. Quantitation of FIPV Replication

Viral RNA was isolated from effusion-derived cell pellets and lymph node tissue using a commercial kit (PureLink RNA Mini Kit, Invitrogen, Waltham, MA, USA) and subsequently treated with DNase (TURBO DNase). Complementary DNA (cDNA) was generated using a commercial kit (High-Capacity RNA-to-cDNA kit, Applied Biosystem, Waltham, MA, USA). Target copy numbers were determined via quantitative reverse-transcription polymerase chain reaction (RT-qPCR) using the QuantStudio 3 Real-Time PCR System and PowerUp SYBR Green Master Mix (Applied Biosystem, Waltham, MA, USA) in a 10 µL reaction as previously described.

PCR primers targeting FIPV were based on a consensus sequence of the feline coronavirus 7b gene. The sequence for the forward primer was 5′-GGAAGTTTAGATTTGATTTGGCAATGCTAG and the sequence for the reverse primer 5′-AACAATCACTAGATCCAGACGTTAGCT. Negative control reactions include a non-template control (molecular grade water) and a reaction excluding reverse transcriptase (RT-). RT-qPCR for the feline GAPDH housekeeping gene was performed in parallel, and the results were normalized to 1 × 10^6^ copies of GAPDH. Quantification of FIPV RNA and GAPDH copy numbers was based on a standard curve generated from viral transcripts prepared through the in vitro transcription of a plasmid (pCR2.1, Invitrogen), as described previously [35].

### 2.7. FIPV Serotype I and II Standard PCR

A nested standard PCR strategy was used to distinguish FIPV serotypes I and II [36]. The reactions utilized published primers with DNA Polymerase (Taq, Invitrogen) following the manufacturer’s protocol for a 50 µL reaction. PCR was performed in an Eppendorf Mastercycler conditions as follows: 94 °C for 5 min followed by 35 cycles of 94 °C for 1 min, 1 min of annealing at 47 °C for the first round of amplification and 50 °C for the nested PCR, and 72 °C for 1 min, followed by a final elongation step of 72 °C for 10 min. The appropriate amplicon size was determined using 1% agarose gel electrophoresis.

### 2.8. S1/S2 PCR Amplification, Cloning and Sequencing

RT-PCR and FCoV *spike* gene-specific primers were used to amplify a region within the viral genome flanking the S1/S2 site [37]. PCR conditions were as described previously with a 50 °C annealing step. The resulting amplicons were assessed via agarose gel electrophoresis, and fragments of the correct size were inserted into a plasmid vector using the TA Cloning Kit and pCR2.1 plasmid vector following the manufacturer’s recommendations (Invitrogen). Plasmids were transformed and amplified in competent *E. coli* bacteria, bacterial clones were selected using antibiotic plates, and plasmids were isolated using Zymo Research’s ZymoPURE Plasmid Miniprep Kit (Genesee Scientific, Morrisville, NC, USA). Three plasmids were subsequently prepared per sample for Sanger sequencing following vendor guidelines (Genewiz, Azenta Life Sciences, Burlington, MA, USA). Spike RNA and amino acid sequences were compared using MacVector software (verion 17.0, ClustalW). Amino acid sequences were analyzed using WebLogo 3.1 software available on the internet (http://weblogo.threeplusone.com/create.cgi (accessed on 1 June 2023)).

### 2.9. Necropsy and Tissue Analyses

If permitted by the animal’s caretaker, cats that succumbed to disease had a timely and complete necropsy examination performed within 24 h of death. The single exception was cat 20, who died 326 days after initiating therapy, and 278 days after completing antiviral therapy. For this animal, the cadaver was frozen by the caretaker, submitted to UC Davis and thawed for 48 h before the necropsy examination. All gross lesions were digitally photographed. A complete set of tissues, including the brain and eye, were collected and immersed in 10% buffered formalin for a minimum of 24 h. Abdominal lymph node tissue was collected in 500 μL RNALater and frozen at −70 °C until utilized (molecular studies). Effusion, if present, was also collected and frozen at −70 °C. Formalin-fixed tissues were trimmed, embedded in paraffin and routinely processed for histological examination (hematoxylin-and-eosin-stained sections). Tissues with pyogranulomatous perivascular inflammation, characteristic of FIP, were further evaluated by an immunohistochemistry (IHC) assay to detect the coronaviral antigen (FIPV3-70, Custom Monoclonals International, Sacramento, CA, USA) [38]. Histology and immunohistochemical stains were performed at the UC Davis Veterinary Histology Laboratory and interpreted by a single anatomic pathologist (BGM). Each IHC stain was performed in parallel with known FIP-positive feline control tissue and an irrelevant isotype-control antibody as the negative control.

### 2.10. Statistical Tests

For normally distributed data, graphical numerical data were presented as the mean of three or more values, with the standard deviation or range represented by error bars. For non-normally distributed data, data were presented as the median of three or more values, with the standard error of the median represented by error bars. Statistical differences were determined by unpaired Student’s *t*-tests. A *p* value of <0.05 was considered statistically significant. Graphs were produced and statistics and linear regression equations performed with Prism 9 software (GraphPad Software, Inc., La Jolla, CA, USA).

## 3. Results

### 3.1. Serology

**Figure 1:**
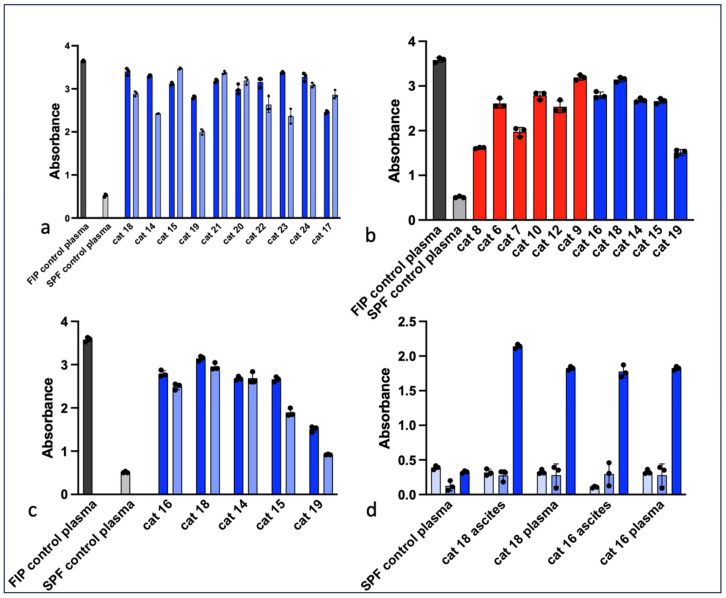
ELISA results for plasma and effusions derived from cats with FIP. (**a**) ELISA absorbance values for paired feline plasma from week 0 (dark blue bars) and week 11 (light blue bars). Data are presented as the mean (or median) of technical triplicates and standard deviation (or standard error of the median). The grey bar represents plasma from a cat experimentally infected with FIPV (positive control) and the light grey bar represents plasma from a specific-pathogen-free cat (negative control) [19]. (**b**) ELISA absorbance values for cats with FIP that died or were euthanized (red bars) and cats that successfully entered remission (blue bars). Controls are as for (**a)**. (**c**) For cats that successfully entered remission, the anti-FCoV response is depicted for each patient using samples derived from ascites effusions (dark blue bars) or plasma (light blue bars). Controls are as for (**a**). (**d**) For cats that successfully entered remission, the isotype-specific anti-FCoV response is indicated for IgM (light blue bars), IgA (med blue) and IgG (dark blue). SPF control plasma serves as the negative control.

The ELISA results for FCoV antibodies in serum, plasma or effusions from cats with FIP are presented in Figure 1. In cats treated with antiviral compounds that entered remission, plasma FCoV antibodies were generally maintained from week 0 to week 11 of treatment (Figure 1a). Figure 1b demonstrates plasma ELISA data for a subset of cats that succumbed to FIP and for cats that successfully entered remission. In the aggregate, the mean ELISA absorbance values were not significantly different between these two populations of cats with FIP (*p* = 0.5847, not significant). In Figure 1c, for samples obtained at time zero before beginning treatment, the plasma FCoV ELISA absorbance values were similar to the values for the paired effusions from each animal. In both plasma and effusions, anti-FCoV antibodies are primarily of the IgG isotype, and not IgM or IgA isotypes (Figure 1d).

### 3.2. Western Blot (Immunoblot)

**Figure 2:**
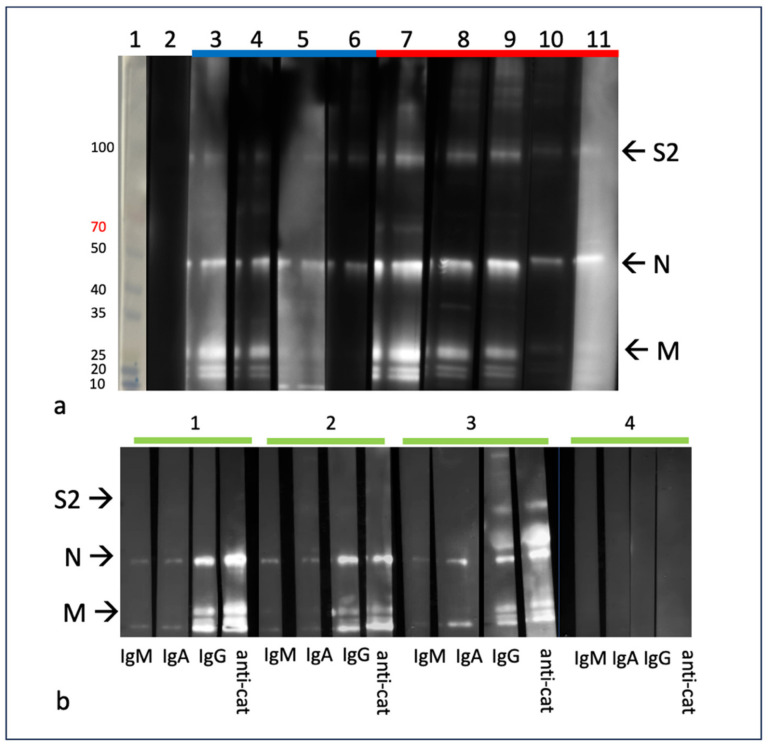
Western blot (immunoblot) assays demonstrating anti-FCoV responses from cats with FIP. FCoV N protein (~43 kDa), M protein (~25 kDa) and S2 (~90 kDa) are labeled with the corresponding letter. (**a**) Western blot assay demonstrating total Ig binding to FCoV proteins for study cats. Cats that successfully entered remission are indicated by the blue bar (lanes 3–6), and cats that died or were euthanized are indicated by the red bar (lanes 7–11). Lane 1—molecular weight markers, lane 2—SPF negative control plasma, lane 3—cat 14, lane 4—cat 15, lane 5—cat 16, lane 6—cat 17, lane 7—cat 9, lane 8—cat 12, lane 9—cat 10, lane 10—cat 7, lane 11—cat 11. (**b**) Western blot assay demonstrating Ig subtype binding for a subset of study cats. Lane 1—cat 18, lane 2—cat 14, lane 4—cat 19, lane 5—SPF feline plasma (negative control).

The representative Western blot results for plasma from cats with FIP are depicted in Figure 2. At least three FCoV proteins can be identified in these immunoblots—the S2 subunits of S (~90 kDa), N (~43 kDa) and M (~25 kDa). Several of the immunoblots also feature a smaller doublet band at approximately 10–20 kDa which may represent the FCoV E protein (8–12 kDa) [39] and the accessory protein 7b (~26 kDa) [40]. Immunoblots using the monoclonal antibodies FIPV3-70 and CCV both reveal a single band at ~43 kDa, consistent with the FIPV N protein.

In Figure 2a, a series of immunoblots utilizing plasma from FIP cats that died during the study and FIP cats that entered remission variably demonstrate bands corresponding to FCoV S2, N and M (all samples from the time of diagnosis). While the band corresponding to FCoV N is recognizable in blots derived from all the FIP study cats, the M and S2 bands vary in intensity. An overt difference in the blot banding patterns between the FIP cats that died and those in remission is not evident. In Figure 2b, the IgG immunoblots have a much stronger signal than the immunoblots corresponding to IgA and IgM, consistent with the ELISA results in Figure 1d, indicating an IgG-predominant anti-FCoV response.

### 3.3. Pathology

**Figure 3:**
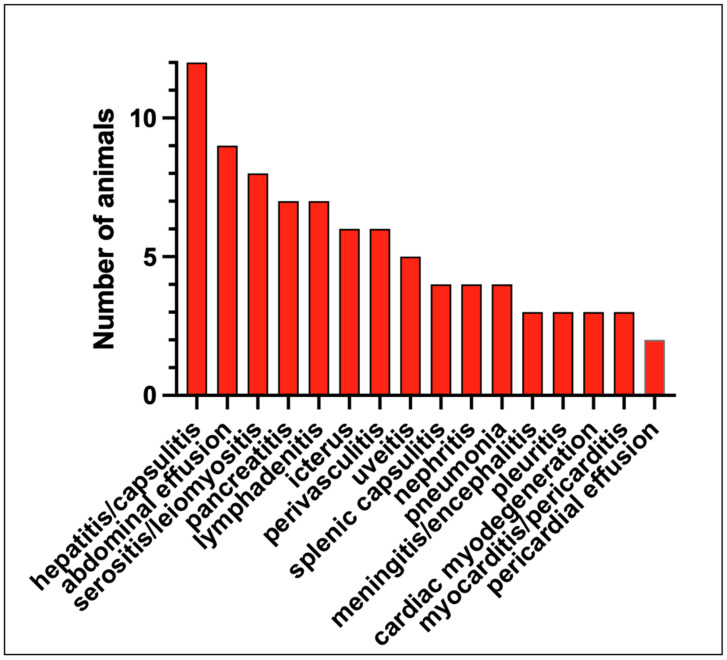
Frequency of pathologic lesions identified in the necropsied cats with FIP.

**Figure 4:**
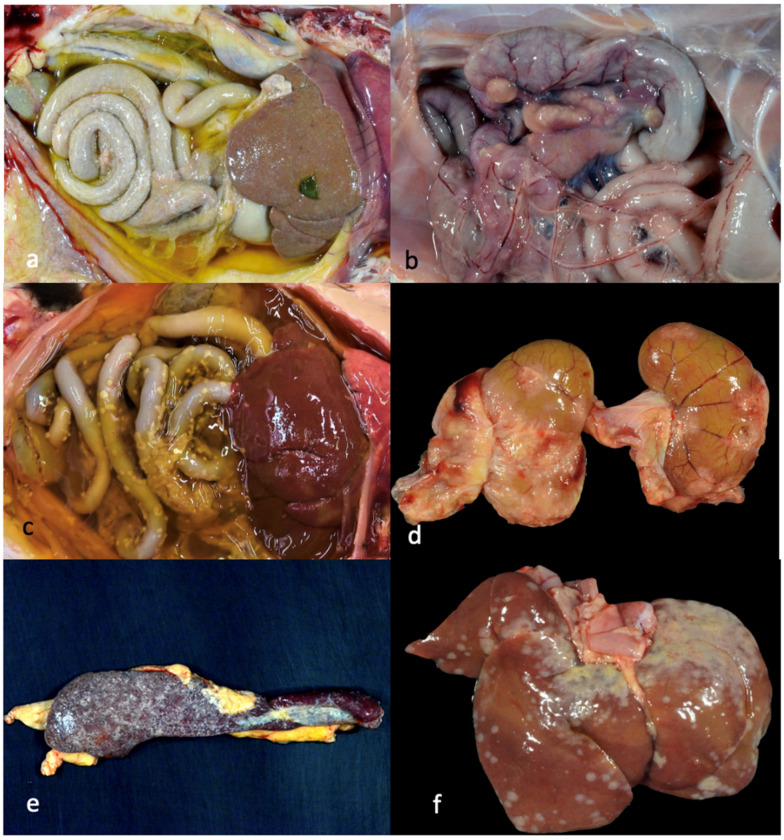
Select gross lesions identified in the necropsied cats with FIP. (**a**) Open abdomen demonstrating icterus, abdominal effusion, fibrinous serositis and hepatic capsulitis (wet/effusive FIP, 4 days of antiviral (AV) treatment, cat 8). (**b**) Open abdomen demonstrating granulomatous mesenteric lymphadenitis/lymphadenomegaly (dry/granulomatous FIP, untreated cat 3). (**c**) Open abdomen and thorax demonstrating abdominal effusion, icterus and serositis (wet/effusive FIP, 3 days of AV treatment, cat 6). (**d**) Bilateral kidneys demonstrating granulomatous nephritis and capsulitis (dry/granulomatous FIP, 6 days of AV treatment, cat 10). (**e**) Spleen demonstrating splenic capsulitis (4 days of antiviral treatment, cat 8). (**f**) Liver demonstrating hepatic capsulitis (1 day of antiviral treatment, cat 4).

**Figure 5:**
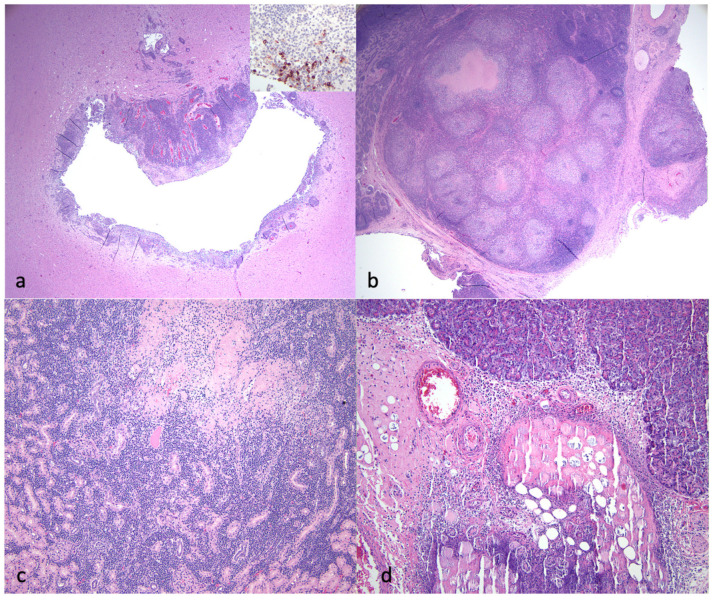
Select histological lesions identified in the necropsied cats with FIP. (**a**) Section of brainstem demonstrating periventriculitis and encephalitis. The inset image demonstrates higher magnification of an IHC for FCoV antigen (+++ viral antigen, untreated cat 3). (**b**) Mesenteric lymph node with necrotizing lymphadenitis lesion. IHC for FCoV demonstrated abundant viral antigen (IHC not shown, untreated cat 3). (**c**) Kidney demonstrating necrotizing interstitial nephritis (6 days of antiviral treatment, cat 5). (**d**) Pancreas demonstrating necrotizing pancreatitis and steatitis (5 days of antiviral treatment, cat 9).

**Figure 6:**
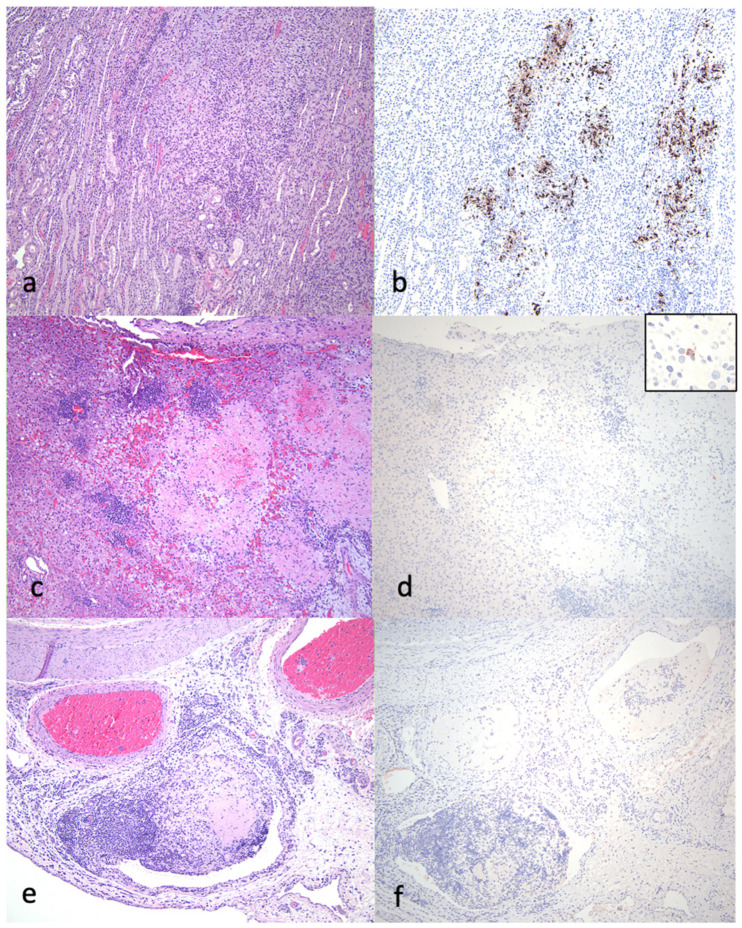
Select histological lesions identified in the necropsied cats with FIP. (**a**,**b**) Kidney demonstrating interstitial nephritis lesion, HE-stained section: (**a**). FCoV IHC of renal section demonstrating abundant (+++) viral antigen; (**b**) untreated cat 2. (**c**,**d)** Liver demonstrating fibrinonecrotizing hepatitis, HE-stained section: (**c**) FCoV IHC of same hepatic section demonstrating minimal (+) viral antigen; (**d**) inset—higher magnification of same section demonstrating single positive macrophage (7 days of antiviral treatment, cat 11). (**e**,**f**) Seven days of antiviral therapy, cat 11. Serosa and mesentery of gut demonstrating fibrinous phlebitis and perivasculitis lesion, HE-stained section (**e**). FCoV IHC of same section demonstrating no (0) viral antigen (**f**).

**Figure 7:**
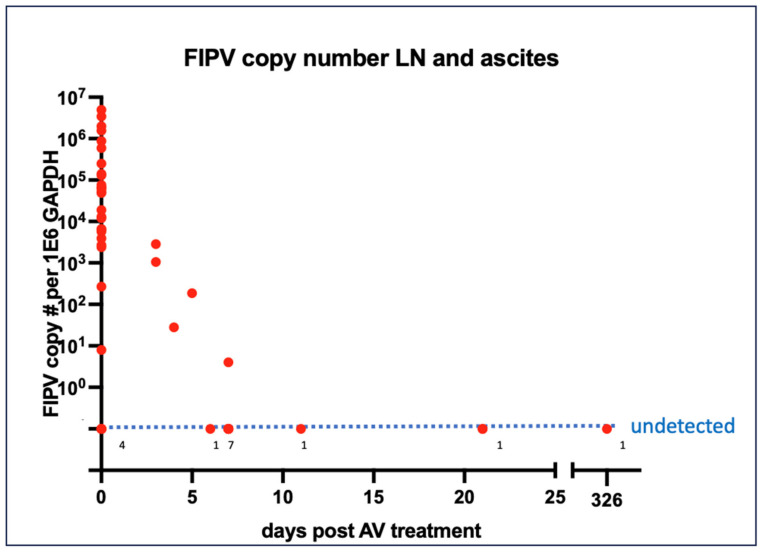
FCoV nucleic acid quantification in lymph nodes and effusions in response to antiviral therapy. Numbers along the “undetected” dotted line refer to the number of cases with no FCoV RNA detected at that specific time point.

Pathologic lesions identified in the 13 study cats that were euthanized or succumbed to disease are listed in Table 1 and Figure 3 and are depicted in Figure 4, Figure 5 and Figure 6. The pathologic lesions identified in cats treated with antivirals (Table 1, yellow boxes) and those that died without antiviral treatment (blue boxes) were similar. The most common pathologic lesions identified, in frequency of occurrence, were hepatitis/capsulitis, abdominal effusion (ascites), serositis/leiomyositis, pancreatitis, lymphadenitis, icterus, perivasculitis and uveitis (Figure 3). These lesions are typical of cats with FIP and are depicted in Figure 5 (gross) and Figure 6 and Figure 7 (histopathology). 

A typical FIP-associated nephritis lesion (Figure 6a), from untreated cat 2, demonstrates abundant FCoV antigens (+++) within the lesion through IHC (Figure 7 and Table 1). Interestingly, although a cat treated with an antiviral compound for 7 days (cat 11) demonstrated inflammatory lesions typical of FIP (hepatitis and mesenteric phlebitis/perivasculitis, Figure 6c,e), the FCoV antigen was minimal to undetectable by IHC (Figure 6d,f and Table 1). In general, the FCoV antigen was difficult to detect in the lesions of FIP cats treated for 4 days or more with antivirals prior to death (Table 1, FCoV IHC). This trend suggests that antiviral therapy effectively reduces the lesion-associated viral burden within days of therapy initiation. 

A cat with FIP that was successfully treated and in clinical remission died unexpectedly 132 days after completing antiviral therapy (cat 12). Gross lesions were minimal and not typical of FIP (hemopericardium). Microscopic inflammatory lesions typical of FIP were not identified, and no FCoV RNA was detected in an abdominal lymph node. Follicular hyperplasia was identified in an abdominal lymph node from cat 12.

Pneumonia was identified in three FIP study cats treated with antivirals and one untreated FIP cat. Pneumonia is not a lesion typical of FIP and suggests possible concurrent bacterial infection. Aggregates of Gram-negative bacteria and scattered Gram-negative coccoid bacteria were identified within the inflamed airways of the untreated cat that succumbed to disease (cat 2). Feline herpesvirus antigen was also identified intralesionally through IHC in cat 2. In cats 4 and 10, aggregates of bacteria and foreign material were identified within the pulmonary bronchi, consistent with aspiration pneumonia. Cat 10 also had a history of force-feeding, known to be a mechanism of aspiration pneumonia. Cat 7 had embolic pneumonia and suppurative hepatitis with intralesional bacteria, suggestive of bacterial sepsis in addition to FIP. Microbial cultures of the pulmonary tissues were not performed. 

Cardiac lesions were identified in five cats (Table 1), including myodegeneration (three lesions) and myocarditis/pericarditis (three lesions), in cats treated with antivirals (Table 1). FCoV IHC assays were performed for three of the cats with cardiac lesions in this study, and no viral antigen was detected (Table 1). 

### 3.4. Virology

Normalized FCoV loads derived from either abdominal lymph node tissue or effusions from 33 cats are plotted in Figure 7 as a function of the sample collection time after initiating antiviral therapy. Within 10 days of initiating therapy, viral loads decreased and became undetectable. A linear regression of these data generated the equation y = −35281 * X + 404476. Solving for the x intercept where the viral load becomes undetectable yielded 11.46 days after the initiation of antiviral therapy. 

**Figure 8:**
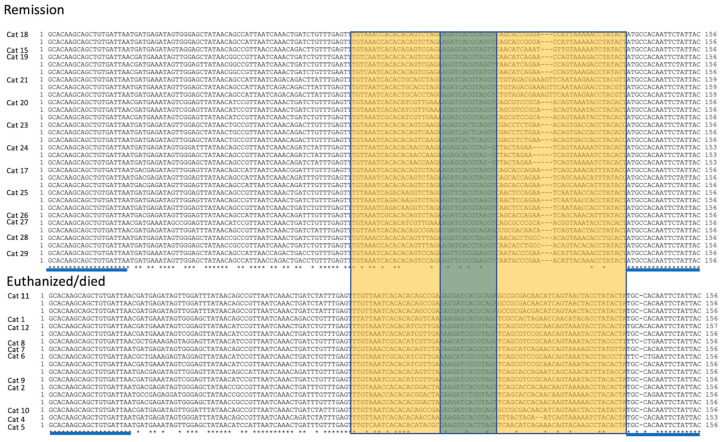
S1/S2 nucleotide sequences for the FCoV spike gene. Sequences derived from study cats that successfully entered remission are listed in the upper block, while those derived from cats that died or were euthanized are listed in the lower block. Individual cat numbers are listed on the left, and 1–3 sequences are depicted per animal as a, b or c. The hypervariable region (yellow) flanks the S1/S2 sequence (green). Primer sites are indicated by blue lines. Asterisks (*) indicate conservation of the nucleotide sequence at that locus.

**Figure 9:**
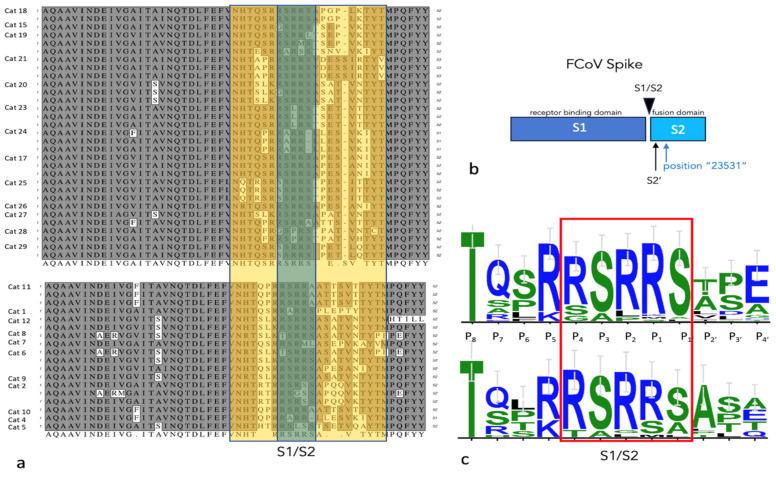
S1/S2 amino acid sequences for the FCoV spike gene. (**a**) Sequences are organized as in Figure 8. (**b**) A schematic of the FCoV S protein demonstrating the S1 and S2 portions and the cleavage sites and locus 23,531. (**c**) The relative frequency of the amino acids at each locus are depicted in this WebLogo 3.1 analysis (http://weblogo.threeplusone.com/create.cgi (accessed on 1 June 2023)). The S1/S2 site is boxed in red.

Twenty-one cats were PCR “serotyped” (genotyped) and had the S1/S2 region of the coronaviral *spike* gene amplified, cloned and sequenced from effusions or abdominal lymph node tissue. The *spike* sequences were consistent with serotype I FIPV (FIPV I/II standard PCR) in all assessed cats. Thirty-one *spike* sequences are displayed for FIP cats treated with antivirals who successfully entered remission, and 18 sequences are displayed for cats that died or were euthanized during the study (Figure 8). Figure 9 demonstrates the amino acid translation for the sequences displayed in Figure 8. The hypervariable region (yellow) and S1/S2 cleavage site (green) are indicated (Figure 8 and Figure 9). 

## 4. Discussion

Over the past 5 years and across the globe, the identification of a small number of effective antiviral therapies have facilitated the remarkably successful treatment of cats with FIP. However, not every treated cat has a favorable outcome. Most of these cats were directly treated by their caretakers, and as a result, detailed virologic, serologic and pathologic outcomes are limited to a relatively small number of published studies. To acquire detailed outcome data, we leveraged multiple, concurrent academic clinical trials at the University of California, Davis, involving 60 cats with naturally occurring FIP and 56 treated with antiviral compounds along with 4 untreated cats. 

Using ELISA and immunoblot assays and feline plasma, serum and effusions obtained throughout the antiviral treatment period, we found that anticoronaviral serologic responses were similar between cats that successfully entered remission and those that succumbed before, during or after the completion of antiviral therapy. That is, we did not identify an overt effect of the antiviral therapies or recovery on the humoral FCoV profile. We found that anti-FCoV humoral responses were robust and maintained throughout the 12 weeks of treatment, primarily comprising the IgG isotype and directed towards the FCoV M and N proteins. For an individual animal, anti-FCoV antibodies within effusions and blood were of similar magnitude and immunoglobulin isotype.

The FCoV Membrane (M) protein is a glycosylated structural protein anchored in the viral envelope [41]. FCoV M is the most abundant viral structural protein and has a size variably defined as 25–30 kDa [39] or 28–32 kDa [42]. Although abundantly distributed throughout the viral envelope, M protein has a relatively small ectodomain, which makes it less antigenic [43]. The FCoV Nucleocapsid (N) protein binds the viral genomic RNA and protects it from degradation [39]. The size of FCoV N has been described as ranging from 43 kDa [42] to 50 kDa [39]. The FCoV spike (S) protein forms a trimer on the viral envelope and is the primary ligand facilitating cell entry. The serotype I FCoV S protein (180–220 kDa [44]) has an S1/S2 cleavage site that is activated by an as yet undefined cell membrane-associated protease [39]. Cleavage of the S protein at this site yields the S1 (receptor binding domain) and S2 (fusion domain) subunits of S. The S2 subunit has been defined as measuring 80–90 kDa in size [42]. A second S cleavage site, S2′, lies within the fusion domain and is present in both viral serotypes [39]. Data suggest that mutations in the S1/S2 and S2′ cleavage sites may be associated with FCoV pathogenesis and the critical FECV to FIPV biotype switch [37,39].

The immunoblots demonstrated antibody–antigen bands consistent with the size of FCoV N, M and S2 proteins and were similar between plasma and effusions. An obvious difference in the banding pattern between cats with successful versus unsuccessful treatment outcomes was not identified. The most robust humoral response was directed against the viral N and M proteins and, to a more limited degree, fragments of the S protein (S2). This somewhat surprising result might be an artifact of the immunoblot detection method, as the wild-type FCoV S sequence is likely to diverge from the target viruses utilized in the immunoblot assay—serotype II (WSU 79-1146) and serotype I (Black I) FIPV. Therefore, antibodies naturally arising against S in an individual cat may or may not bind to the culture-adapted FCoV S proteins utilized in the assay. As a result, a portion of the humoral anti-S response may have gone undetected in these assays. 

Pathologic lesions identified in the 13 necropsied cats were generally typical of cats with the effusive “wet” or granulomatous “dry” forms of FIP. Using immunohistochemistry, the FCoV antigen was readily detected in multiple inflammatory lesions from cats that had not been treated, or had been briefly treated (1–2 days) with antiviral therapy. The viral antigen became progressively more difficult to identify as a function of treatment duration. This treatment-associated difficulty in identifying the viral antigen has been previously reported for cats treated with GS-441524 that eventually succumb to disease [18].

We also identified multiple pathologic lesions not typically considered to be related to FIP, including pneumonia and cardiac-associated lesions such as myocarditis and cardiac myodegeneration. Some of these naturally infected cats had intercurrent diseases, like bacterial sepsis or aspiration pneumonia, which likely contributed to their demise. Most of the cats that died after beginning antiviral therapy died soon after therapy commenced, within days of beginning antiviral therapy. This temporal association with death soon after initiating antiviral therapy has been previously reported [18,26] and suggests that several of the cats that entered into the clinical trials had “advanced disease”, with poor nutritional balance, severe multiorgan disease, high viral burden or concurrent disease.

Although no viral antigen was detected by IHC in the cardiac tissue of three cats with myocardial lesions, this does not prove that FCoV played no direct role in myocardial disease. Several previous reports have described the occurrence of cardiac disease in cats with FIP treated with antivirals [18,45], and myocarditis has been associated with SARS-CoV-2 infection in human patients [46]. At present, the pathogenesis of these lesions and the relationship of FCoV and antiviral compounds remain undetermined. 

In a recent peer-reviewed study, a cat with molecularly confirmed ocular FIP (uveitis) was successfully treated, and then, died from a road traffic accident 164 days after 84 days of antiviral treatment with oral GS-441524 [32]. The only identified lesion, other than acute trauma, was lymphadenomegaly. Neither FCoV RNA nor an antigen was detected in any tissue, including feces. These findings are in alignment with cat 13 from our study, who died unexpectedly 132 days after completing antiviral therapy. These two cases indicate that effective antiviral treatment can cure cats of FIP and effectively eliminate FCoV viral RNA and antigen from the tissues.

In abdominal effusions and tissue samples obtained from 33 of the cats, we identified a rapid reduction in the FCoV viral load in response to the duration of antiviral therapy, consistent with the findings for viral antigen. In a recent study of 12 naturally infected cats with FIP, FCoV was also not detectable by quantitative RT PCR in blood samples after 7 days of antiviral treatment [30]. These data support the in vivo efficacy of the antiviral treatments. 

Studies have indicated that at least three genetic loci in the FCoV *spike* gene may be associated with either the FECV-to-FIPV biotype switch or viral pathogenesis. These include the S1/S2 cleavage site [37], S2′ cleavage site [39] and FCoV genomic position 23,531 [1]. We amplified, cloned and sequenced the S1/S2 region of the FCoV *spike* gene in 21 cats and determined that all viruses were serotype I. Although some genetic variability was identified, both groups of cats that died or were euthanized and cats that successfully entered remission demonstrated the canonical RSRRS amino acid sequence at the S1/S2 cleavage site. We did not identify an obvious difference in the S1/S2 sequence between these two groups of cats. The S1-S2 site in the FCoV S protein is a sequence-dependent furin cleavage site [37]. Serotype I FECV isolated from feces has a highly conserved furin cleavage motif at S1/S2: (R)^P4^-(S > A)^P3^-(R)^P2^-(R)^P1^-(S)^P1^′, or R S/A R R S [37]. FCoV isolated from cats with FIP has consistently mutated sites at positions P2 (S, H, P, L) and P1 (M, G, T) [37]. In our sequencing data, cats entering remission had uncommon mutations at position P2 (L, P) and rare mutations at P1 (S, M), while cats that died had rare mutations at P2 (G, L) and P1 (S, M). The biological relevance of these minor substitutions in the S1/S2 sequence was not determined.

The limitations of this study include the incomplete data set for serology and virology samples. Unfortunately, biological samples were not available for all the cats involved in the study. The inclusion of cats in the study with intercurrent disease (bacterial sepsis and pneumonia) or those with advanced disease at presentation may have complicated the determination of antiviral efficacy. Another limitation was the failure to amplify and sequence the entire FCoV *spike* gene to interrogate other genetic loci. Ultimately, further studies are needed to better understand the risk factors for succumbing to FIP after the initiation of antiviral therapy.

## Figures and Tables

**Table 1:** Postmortem pathology for cats from 4 clinical trials.

	Abdominal Effusion	Icterus	Serositis or Leiomyositis	Vasculitis/Uerivasculitis	Hepatitis/Capsulitis	Nephritis	Lymphadenitis	Splenic Capsulitis	Pancreatitis	Meningitis/Encephalitis	Uveitis	Cardiac Myodegeneration	Myocarditis/Pericarditis	Pneumonia	Pericardial Effusion	Pleuritis
**74-80-41**	**√**		**√** **+++**		**√** **++**		**√** **++**		**√** **0**	**√** **0**						**√** **0**
**74-95-21**	**√**	**√**		**√**	**√**	**√** **+++**			**√**		**√** **+++**			**√**		
**75-63-32**			**√** **++**		**√**		**√** **++**		**√**	**√** **+++**						
**75-14-64** **1d**			**√** **+++**	**√**	**√** **+++**		**√** **+++**	**√** **+++**					**√** **0**	**√**		
**74-89-23** **2d**	**√**	**√**	**√** **++**		**√**	**√** **+++**		**√** **+++**				**√**				
**74-90-61** **3d**	**√**	**√**	**√**	**√**	**√**		**√**		**√**							
**74-84-08** **3d**	**√**		**√** **+**		**√** **0**			**√** **0**	**√** **+**		**√** **0**	**√**		**√**		
**74-83-16** **4d**	**√**	**√**	**√** **0**	**√** **0**	**√** **0**		**√** **0**	**√** **0**								
**74-93-52** **5d**	**√**	**√**			**√**	**√** **0**	**√**		**√**		**√**	**√**	**√** **0**			**√**
**74-98-25** **6d**	**√**	**√**			**√**	**√**		**√**		**√**	**√**			**√**	**√**	**√**
**74-81-36** **7d**	**√**		**√** **+**	**√** **0**	**√** **+**		**√** **0**		**√** **0**							
**74-82-32** **11d**				**√**	**√**						**√**		**√** **0**			
**74-85-67** **132d**															**√**	

Table legend: Blue boxes indicate cats that are untreated, while yellow boxes indicate cats treated with antiviral compounds. Grey boxes indicate FCoV immunohistochemistry (0, +, ++, +++). A “check mark” indicates that the lesion was identified in that animal. The letter “d”, as in “5d”, indicates that the cat died that number of days after beginning antiviral therapy. FCoV IHC findings: 0 antigen not detected; + rare single positive cells; ++ small clusters of positive cells (less than 10 cell aggregates); +++ >large clusters of positive cells (more than 10 cell aggregates).

## Data Availability

The data presented in this study are available on request from the corresponding author.

## Supplementary Materials

The following supporting information can be downloaded at: https://www.mdpi.com/article/10.3390/v16030462/s1, Table S1: FIP Supplemental SS.
